# COVID-19 and Senotherapeutics: Any Role for the Naturally-occurring Dipeptide Carnosine?

**DOI:** 10.14336/AD.2020.0518

**Published:** 2020-07-23

**Authors:** Alan R Hipkiss

**Affiliations:** Aston Research Centre for Healthy Ageing (ARCHA), Aston University, Birmingham, B4 7ET, UK

**Keywords:** carnosine, acetyl-carnosine, inflammation, virus, olfaction, lungs, aging

## Abstract

It is suggested that the non-toxic dipeptide carnosine (beta-alanyl-L-histidine) should be examined as a potential protective agent against COVID-19 infection and inflammatory consequences especially in the elderly. Carnosine is an effective anti-inflammatory agent which can also inhibit CD26 and ACE2 activity. It is also suggested that nasal administration would direct the peptide directly to the lungs and escape the attention of serum carnosinase.

It has become clear that, compared to the young, elderly humans are especially susceptible to fatal infection by COVID-19, most likely due, it is thought, to pre-existing, age-related, underlying conditions. Consequently, a number of recent publications [1,2,3.4] have discussed how the processes of ageing might enhance the impact of the COVID-19 virus upon human physiology. Furthermore, it has been proposed that agents which suppress ageing onset and/or development, should be explored for mitigation of COVID-19 toxicity and mortality in the elderly [1,3,4]. Indeed, a search for effective senotherapeutics has considered a range of possible anti-aging agents and the mechanistic routes that might ameliorate age-related change [3]. It is proposed here that the naturally-occurring dipeptide carnosine (beta-alanyl-L-histidine) should also be considered as a potential senotherapeutic which may help to suppress COVID-19 toxicity.

## Carnosine, aging and COVID-19

There is evidence, first obtained more than 25 years ago, that carnosine delays senescence, extends lifespan and even rejuvenates cultured human lung fibroblasts [5]. Subsequent studies revealed that carnosine (i) can delay ageing and/or related phenomena in some animal models [6-10], (ii) possesses anti-oxidant and anti-glycating activities [11-15], (iii) possesses anti-inflammatory properties [16-20], (iv) is protective towards lung injury [21-23], (v) can decrease the infectivity of RNA viruses Zika, Denge [24] and influenza [25], (vi) is an inhibitor of CD26 (also known and dipeptidyl peptidase IV (DPP4) [26,27] and ACE-2 (angiotensin converting enzyme 2) [28,29], i.e. the cell receptors to which COVID-19 attaches to ensure infection [30,31], (vii) can complex with zinc ions to form polaprezinc [32]; it has been reported that zinc ions can inhibit COVID-19 RNA polymerase thus suppressing viral replication [33].

## Carnosine and blood

Another role for carnosine could include beneficial effects in blood. Recent findings suggest that COVID-19 infection can promote in some patients a hyper-coagulatory state, which is associated increased mortality [34]. The glycolytic by-product methylglyoxal (MG) is generated in increased amounts in type-2 diabetics, especially in erythrocytes following high glycemic-index diets [35]. MG is responsible for much post-synthetic protein glycation in diabetics, including the anti-coagulants anti-thrombin III [36] and plasminogen [37], resulting in a hyper-coagulatory state. Given that carnosine can inhibit protein glycation and possibly scavenge MG, the dipeptide might suppress the MG-induced anti-coagulant modification.

Recent studies have shown that carnosine and acetyl-carnosine are present in human blood; carnosine is present in erythrocytes and acetyl-carnosine is mostly in serum; erythrocytes normally contain carnosine and 10-fold less acetyl-carnosine, while the situation is reversed in serum where acetyl-carnosine is the predominant form. Importantly, these levels decline in elderly humans [38] and low levels of acetyl-carnosine are associated with enhanced frailty [39]. Low carnosine levels have also been detected in blood from patients suffering from age-related macular degeneration [40].

It is perhaps interesting to note that carnosine synthesis may be affected by trauma, even in the distant past, which could influence an individual’s blood carnosine and acetyl-carnosine levels. In adults subjected to childhood trauma, the carnosine synthase gene showed increased methylation, which would decrease carnosine synthesis [41]. Childhood trauma has been linked to psychopathology, accelerated aging-related DNA methylation [42] and schizophrenia [43]. Furthermore, erythrocytes from schizophrenics show evidence of increased aging-related protein glycation [44], while dietary carnosine supplementation has been shown to produce beneficial effects in schizophrenics [45], possibly due, in part, to carnosine’s anti-glycating activity [11,12].

## Carnosine as a possible senotherapeutic

All the factors outlined above, lead one to suggest that carnosine should be considered as a possible senotherapeutic, acting either prophylactically by helping to suppress the development of an increasingly frail senescent phenotype and/or therapeutically by interfering with COVID-19 infection (given its reported effects on DPP4 and ACE2). Furthermore, carnosine’s anti-inflammatory effects may help suppress post-infection inflammatory response and fibrosis [46]. It may also be significant that loss of a sense of smell frequently accompanies COVID-19 infection [47], and that the olfactory lobe is normally enriched with carnosine [48]. Consequently, it is suggested that the dipeptide is worth exploring for suppression of COVID-19 toxicity in aged humans, especially in subjects already possessing age-related dysfunction.

As noted above, a decreased serum level of acetyl-carnosine (unsusceptible to hydrolysis by serum carnosinase) has recently been noted as an indicator of frailty in elderly humans [39], thus it may be informative if blood levels of acetyl-carnosine and carnosine could be determined in COVID-19 infected patients.

There have been a number of studies in which dietary supplementation with carnosine has been explored. Beneficial effects have been observed in schizophrenics [45], Gulf War veterans [49], obese humans [50], diabetics [51], elderly human verbal memory [52], autistic children [53,54] and patients with chronic heart failure [55]. Unfortunately, a major problem with oral administration of carnosine is the presence of carnosinase in serum which will destroy the peptide; indeed high carnosinase activity can limit the efficacy of orally administered carnosine towards chronic kidney disease [56]. An alternative approach would be to use nasal delivery, either as an aerosol or in powder form, thus eliminating the serum carnosinase problem and targeting carnosine directly to the lungs. Hopefully this would suppress COVID-19 infectivity via the dipeptide’s inhibitory effects on both ACE2 and MPP4. Moreover, carnosine’s well-recognised anti-inflammatory activity could suppress any excessive inflammatory response following infection. Such an approach may be especially important in frail subjects, exhibiting low levels of acetyl-carnosine (resistant to carnosinase attack). The precise relationship between these two molecules remains to be revealed; for example, is red cell carnosine a precursor of acetyl-carnosine which is then released from the cell?

## Conclusion.

Carnosine has previously been described as enigmatic [57], and the dipeptide has been shown to exert a variety of effects at biochemical and physiological levels resulting in a large number of possible biological functions [58]. Indeed, as aging is multifactorial, any putative anti-aging agent might be expected to be pluripotent in its actions [59]. Thus, carnosine’s cumulative multifunctional activities could result in a physiologically beneficial outcome in COVID-19 infected aged humans. In order to decrease virus-associated mortality, it is suggested that elderly subjects with low blood carnosine and acetyl-carnosine levels should receive exogenous carnosine, administered nasally.
